# Comprehensive derivation of bond-valence parameters for ion pairs involving oxygen

**DOI:** 10.1107/S2052520615016297

**Published:** 2015-09-30

**Authors:** Olivier Charles Gagné, Frank Christopher Hawthorne

**Affiliations:** a Geological Sciences, University of Manitoba, 125 Dysart Road, Winnipeg, Manitoba R3T 2N2, Canada

**Keywords:** bond-valence parameters, bond-valence equations, the valence-sum rule

## Abstract

Published two-body bond-valence parameters for cation–oxygen bonds have been evaluated *via* the root mean-square deviation (RMSD) from the valence-sum rule for 128 cations, using 180 194 filtered bond lengths from 31 489 coordination polyhedra. Values of the RMSD range from 0.033–2.451 v.u. (1.1–40.9% per unit of charge) with a weighted mean of 0.174 v.u. (7.34% per unit of charge). The set of best published parameters has been determined for 128 ions and used as a benchmark for the determination of new bond-valence parameters in this paper. Two common methods for the derivation of bond-valence parameters have been evaluated: (1) fixing *B* and solving for *R*_o_; (2) the graphical method. On a subset of 90 ions observed in more than one coordination, fixing *B* at 0.37 Å leads to a mean weighted-RMSD of 0.139 v.u. (6.7% per unit of charge), while graphical derivation gives 0.161 v.u. (8.0% per unit of charge). The advantages and disadvantages of these (and other) methods of derivation have been considered, leading to the conclusion that current methods of derivation of bond-valence parameters are not satisfactory. A new method of derivation is introduced, the GRG (generalized reduced gradient) method, which leads to a mean weighted-RMSD of 0.128 v.u. (6.1% per unit of charge) over the same sample of 90 multiple-coordination ions. The evaluation of 19 two-parameter equations and 7 three-parameter equations to model the bond-valence–bond-length relation indicates that: (1) many equations can adequately describe the relation; (2) a plateau has been reached in the fit for two-parameter equations; (3) the equation of Brown & Altermatt (1985 ▸) is sufficiently good that use of any of the other equations tested is not warranted. Improved bond-valence parameters have been derived for 135 ions for the equation of Brown & Altermatt (1985 ▸) in terms of both the cation and anion bond-valence sums using the GRG method and our complete data set.

## Abstract 2

Published bond-valence parameters for cation–oxygen bonds are evaluated with regard to their agreement with the valence-sum rule, and new bond-valence parameters are derived for 135 cations bonded to oxygen.

## 1. Introduction   

Many people have investigated correlations between deviations from Pauling’s second rule (Pauling, 1929 ▸) and bond-length variations in crystals (*e.g.* Baur, 1970 ▸, 1974 ▸; Donnay & Allmann, 1970 ▸; Pyatenko, 1972 ▸; Brown & Shannon, 1973 ▸; Ferguson, 1974 ▸), generally developing quantitative relations between bond length and the strength of a bond. During the 1960s and early 1970s, the term ‘bond strength’ was used, but was later changed to ‘bond valence’ to distinguish these values from Pauling bond strengths. In the early 1970s, several different forms of the (inverse) relation between bond valence and bond length were used, but the equation of Brown & Altermatt (1985 ▸) was eventually accepted as the general form of the bond-valence–bond-length curve: 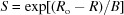, where *S* is the bond valence (in valence units), *R* is the observed bond length, and *R*_o_ and *B* are fitted constants called bond-valence parameters. Brown & Altermatt (1985 ▸) gave values of *R*_o_ and *B* for 141 pairs of ions, and Brese & O’Keeffe (1991 ▸) gave analogous values for 969 pairs of ions. Many smaller-scale studies have produced bond-valence parameters for a wide range of ion pairs that have been compiled by Brown (2002 ▸, 2009 ▸, 2013 ▸). Brown’s latest list of published bond-valence parameters (Brown, 2013 ▸) contains 1749 sets of bond-valence parameters for the equation of Brown & Altermatt (1985 ▸), for 1350 unique ion pairs, and counts 340 sets of bond-valence parameters for 194 cations bonded to oxygen. Several sets of bond-valence parameters are often available for unique ion pairs, and there has been little comparison between different sets of parameters available to determine which is the most suitable for a given ion pair. Here we consider bond-valence parameters for cations bonded to oxygen. Notably, with regard to the bond-valence parameters currently available:

(1) There is no consistency between parameters from different sources; in particular, the criteria used to select the bond lengths used in the derivation of the bond-valence curves vary widely.

(2) Different fitting methods have been used by different authors to derive the bond-valence parameters (*i.e.* there is no consensus on the best way to derive the bond-valence parameters).

(3) Very few alternative forms of the bond-valence–bond-length relation have been tested.

Here, we (1) evaluate published bond-valence parameters for 128 cations bonded to oxygen, using a very large set of bond lengths that have undergone rigorous filtering; (2) investigate many alternative algebraic forms of the bond-valence—bond-length relation; (3) evaluate different fitting methods used in the derivation of bond-valence parameters; (4) determine new bond-valence parameters for 135 cations bonded to oxygen.

## 2. Experimental bond lengths used in this work   

As part of other work examining the dispersion of bond lengths in inorganic crystals, we have used the Inorganic Crystal Structure Database (ICSD) to extract bond lengths for all atoms of the periodic table of elements bonded to oxygen, as a function of oxidation state and coordination number. The following selection criteria were used during collection of the bond-length data: (1) publication date ≥ 1975; (2) *R*_1_ ≤ 0.06; (3) the site of interest is fully occupied by the cation; (4) all bonds involve ions at fully occupied sites; (5) the cation and anion sites of interest show no positional disorder; (6) crystallographic data were measured at ambient conditions; (7) no data from powder, electron or synchrotron diffraction were included; (8) where there was severe ambiguity as to the correct coordination number, the data were not included to avoid error; (9) for H, only neutron-diffraction data were collected.

Following collection of the bond distances, the bond-length distributions were examined for outliers. Where outliers were identified, the original publications were examined to validate the distances or identify errors. The most common source of error involved sites which, in the ICSD, were erroneously identified as containing only one cation whereas inspection of the original paper showed that cation disorder was present (*e.g.* for Si, large mean distances commonly involved the presence of Al^3+^ at the Si^4+^ site, and small distances involved the presence of B^3+^ at the Si^4+^ site). We note here that verified outliers that showed no apparent error were retained in our analysis. Where such analysis had been done for specific ions, we checked our results with those given previously to ensure compatibility (or confirm the validity of any differences). For example, Sidey (2013 ▸) gives the shortest [3]-coordinated B^3+^—O distance as 1.20 Å, in close accordance with our value of 1.22 Å, and Mills *et al.* (2009 ▸) and Mills & Christy (2013 ▸) use maximum Sb^3+^ and As^3+^ distances of 3.5 Å, in reasonable accordance with our values of ∼ 3.4 Å. Use of the above criteria resulted in 180 369 bond lengths from 31 521 coordination polyhedra, for 135 ions bonded to oxygen from 9367 refined crystal structures.

## 3. Method of evaluation of bond-valence parameters   

To evaluate the bond-valence parameters for an ion pair, we calculated the root-mean-square deviation (RMSD) between the bond-valence sum (using the bond-valence parameters and the experimental bond lengths) and the valence of the constituent cation for each polyhedron, over the entire dataset of coordination polyhedra for that cation
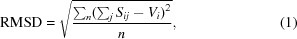
where 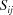 is the bond valence between ions *i* and *j*, 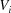 is the valence of the *i*th cation, and the sum is over the 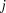 bonds that cation *i* makes to O for the *n* coordination polyhedra available from the dataset of that particular ion pair. This method evaluates deviations from the valence-sum rule (Brown, 2002 ▸), and is applicable to any parameterization. From here on, any mention of RMSD in the text will imply the deviation to be from the valence-sum rule, in valence units (v.u.).

Brown & Shannon (1973 ▸) reported the *relative* RMSD (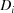), on the basis of a unit of charge
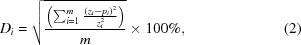
where 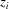 is the valence of ion *i*, 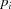 is the bond-valence sum, and *m* is the number of ions of type *i*. This expression has been used by many people reporting new bond-valence parameters. However, the basis of bond-valence curves is the valence-sum rule (Brown, 2002 ▸), and minimization of deviations from the valence-sum rule involves bond valences, not bond valences divided by valence, and hence a more appropriate measure of agreement with the valence-sum rule involves equation (1) rather than equation (2). Although equation (1) is the recommended way of reporting the RMSD in the future, our results will be reported using both equations (1) and (2) for the most part throughout this work, so that they can easily be compared with other published work.

## 4. Evaluation of published oxide bond-valence parameters   

We evaluated 244 pairs of bond-valence parameters (*R*_o_, *B*) for 128 ion pairs involving cations bonded to O^2−^. By and large, bond-valence parameters have been, and continue to be, derived based on the first coordination shell of ions. However, Adams (2001 ▸, 2014 ▸) used the concept of bond softness to argue for the consideration of higher coordination shells in the determination of bond-valence parameters (which he calls softBV parameters) for use in dynamic situations where the use of discrete coordination number is not continuously applicable (*e.g.* ionic conduction; Adams & Prasada Rao, 2014 ▸). Due to the coordination-based nature of our dataset, we did not evaluate softBV parameters.

Table S1 of the supporting information gives the bond-valence parameters of the constituent ions and their associated RMSD obtained from equation (1), listed in the same order as in Brown (2013 ▸), using the same reference codes. The RMSD values range from 0.033 to 2.451 v.u. However, the extremely large values are caused by inappropriate parameters; for example, Cm^3+^ has two published sets of parameters, with RMSD values of 1.500 and 0.161 v.u., and U^6+^ has three sets of parameters with RMSD values of 0.894, 0.699 and 0.193 v.u., respectively. The mean value of the RMSD for all published parameters using our dataset is 0.219 v.u. with a standard deviation of 0.232 v.u. and a median value of 0.241 v.u.

We note here that it is critical for bond-valence parameters to be evaluated in the same way they were derived; while this may seem intuitive, we often observed poor agreements for ions showing large gaps in their bond-length distributions (*i.e.* ions that form ‘secondary bonds’), as different sets of bond-valence parameters available for the same ions were presumably derived both with and without the inclusion of secondary bonds (*e.g.* for I^5+^, Te^4+^). Following experimentation with this practice, we conclude that the inclusion of the long bonds in the first coordination shell leads to better bond-valence sums, and have therefore retained them in our dataset for this evaluation. As a corollary, as we derive our bond-valence parameters (below) using the longer bonds where appropriate (*e.g.* elements of periods 4–6, typically not transition metals), the longer bonds should be included when using the parameters derived in this work.

From the results of Table S1, we may identify a set of best published parameters that provides a useful benchmark for comparison in the derivation of new bond-valence parameters.

## 5. The H atom   

It is necessary to treat the H atom somewhat differently from the other atoms of the periodic table for two reasons: (1) for H atoms, positional parameters derived from X-ray data show significant systematic error, as the electron density notionally associated with the H atom is partly delocalized into the O—H bond, leading to O—H distances that are systematically shorter than the O—H internuclear distances. In turn, this will lead to H⋯O (hydrogen-bond) distances that are systematically longer than the H⋯O internuclear distances; (2) some authors suggest the use of more than one pair of bond-valence parameters to model the relation for this atom. These conditions are specified in Table S1 for each reference.

Grabowski (2000 ▸) used neutron-diffraction data to derive a single pair of parameters, *R*_o_ = 0.93 Å and *B* = 0.40 Å, resulting in a RMSD of 0.035 v.u. for our dataset. Also using neutron-diffraction data, Brown (2002 ▸) proposed the use of three pairs of parameters to model the relation over specific ranges of bond lengths (the resulting RMSD for our dataset is 0.059 v.u.) and argued that the use of different parameters over different bond-length ranges gives better sums around the O^2−^ ions than the parameters of Alig *et al.* (1994 ▸).

Yu *et al.* (2006 ▸) argued that hydrogen requires two sets of parameters, one set for *s* > 0.5 v.u. (the donor–hydrogen bond), and another set for *s* < 0.5 v.u. (the hydrogen–acceptor bond); they also give 1.30 Å as the cut-off between stronger and weaker bonds. Although they do not specify if they used X-ray data, neutron data or a combination of both, the reported bond lengths strongly suggest the sole use of X-ray data. As a result, their parameters are not directly compatible with our dataset, which consists of neutron-diffraction data for hydrogen. However, we decided to test their parameters on our dataset of 224 coordination polyhedra for hydrogen, to evaluate the effect of using X-ray *versus* neutron data for H^+^; we used their first set of parameters (*R*_o_ = 0.79 Å and *B* = 0.37 Å) with the shorter of the O—H distances and their second set of parameters (*R*_o_ = 1.409 Å and *B* = 0.37 Å) with the longer of the H⋯O distances, which resulted in an overall RMSD of 0.181 v.u.

The lowest RMSD for bonds involving hydrogen and oxygen (0.035 v.u.) is thus obtained for the single pair of parameters of Grabowski (2000 ▸), and suggests that a single pair of parameters is sufficient to deal with bonds involving hydrogen and oxygen.

## 6. Use of bond-valence parameters for hydrogen–oxygen bonds   

Bond-valence parameters derived from neutron-diffraction data (such as those we give later) are obviously not relevant to hydrogen positions from unconstrained refinement of X-ray diffraction data (for the reasons outlined above). However, most information on hydrogen in crystal structures originates from X-ray diffraction data. The best way around this situation is to use constrained refinement in the derivation of hydrogen positions. The O_donor_—H distance may be softly constrained to an appropriate value (∼ 0.96–0.98 Å) for OH and H_2_O groups involved in asymmetric hydrogen bonds, and the H—H distance in H_2_O groups may be constrained to ∼ 1.55 Å (which gives an H—O—H angle of ∼ 105°). Of course, the result is only approximate, but the ensuing H⋯O_acceptor_ distances are likely to be far closer to the analogous nucleus–nucleus distances than those derived by unconstrained X-ray refinement. This method of refinement allows the use of bond-valence parameters derived from neutron-diffraction data with bond lengths derived from X-ray diffraction data, and usually leads to good bond-valence sums.

## 7. Comments on fixing the *B* parameter   

The results of the evaluation (Table S1) give us some insight into the practice of fixing the *B* parameter (to 0.37 Å), an issue that has received some comment in recent years (Adams, 2001 ▸; Krivovichev & Brown, 2001 ▸; Locock & Burns, 2004 ▸; Sidey, 2008 ▸, 2010 ▸; Brown, 2009 ▸, 2014 ▸; Mills *et al.*, 2009 ▸; Krivovichev, 2012 ▸). Many ions have bond-valence parameters to oxygen available for both fixed and refined values of *B*, and we may use these ions to evaluate the effectiveness of fixing *B* to 0.37 Å. Out of 37 instances, 12 ions have lower RMSD values for *B* = 0.37 Å, whereas 25 ions have lower RMSD values for *B* ≠ 0.37 Å. This comes as a surprise, as fitting with two variable parameters should give at least as good a fit as fitting with only one variable parameter. This result is probably due to the choice of method for the derivation of the bond-valence parameters, as this can greatly influence the quality of the fit; the use of a poor method of derivation that allows refinement of both *R*_o_ and *B* can easily lead to a poorer fit than the method of fixing *B*, as will be shown later. Nonetheless, significant improvements in fit with two variable parameters are common. A persuasive example is that of Burns *et al.* (1997 ▸). Their parameters for U^6+^ have *B* = 0.519 Å and result in a RMSD value of 0.158 v.u. for our dataset (585 polyhedra), whereas the other parameters (with *B* = 0.37 Å) give a RMSD of 0.690 and 0.889 v.u. (Table S1). Even where bond-valence parameters with *B* = 0.37 Å give low RMSD values, the fit can be improved significantly by allowing *B* to vary. For example, Mills & Christy (2013 ▸) derive new parameters for Te^6+^ with *B* = 0.56 Å, resulting in a RMSD value of 0.146 v.u. compared with 0.229 v.u. for the available parameters with *B* = 0.37 Å). These examples suggest that both *R*_o_ and *B* should be varied in the derivation of bond-valence parameters; of the 244 pairs of bond-valence parameters examined here, 191 have *B* fixed at 0.37 Å.

## 8. Comments on the level of fit   

The mean RMSD for the 244 pairs of bond-valence parameters evaluated here, weighted by the number of coordination polyhedra of the ions, is 0.174 v.u. [7.34% per unit of charge using the equation of Brown & Shannon, 1973 ▸; equation (2)]. The set of best parameters available for each ion (the 128 best pairs) has a mean weighted-RMSD of 0.136 v.u. (5.68% per unit of charge). These values are slightly higher than those commonly reported in the literature, and to the generally accepted ‘5% error margin’ observed by Brown & Shannon (1973 ▸). This difference may be due to the fact that authors typically select a small subset of ‘high-quality’ structures from the available data to derive bond-valence parameters, the size of which strongly influences the reported RMSD value, which in turn does not necessarily reflect the fit for all data. Although the data we use here have been thoroughly filtered for errors, our derivation of the bond-valence parameters (below) foregoes this practice to reduce the possibility of such bias.

From the evaluation of the published bond-valence parameters, we conclude that the fit of the currently available parameters to the valence-sum rule is variable and can be significantly improved.

## 9. Parameterization   

### 9.1. Methods of derivation of the bond-valence parameters   

Bond-valence parameters have been derived using a variety of methods based on different optimization criteria for both experimental and extrapolated data. Here we discuss the most common methods used in the derivation of bond-valence parameters based on experimental data.

#### 9.1.1. Least-squares fitting   

The initial form of the bond-valence equation proposed by Brown & Shannon (1973 ▸) is
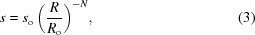
where 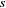 is the bond valence (called bond strength by them), *R* is the bond length, 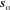 is a parameter usually set to have 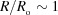, and 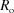 and *N* are the bond-valence parameters. Brown & Shannon (1973 ▸) derived their bond-valence parameters in three different ways using least-squares fitting to minimize deviations from the valence-sum rule:

(1) vary 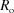 and *N* for the incident bond-valence sums around the cations;
(2) fix *N* and vary 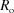 for the incident bond-valence sums around the cations;
(3) vary 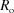 and *N* for the incident bond-valence sums around the cations and the anions.

In principle, method (3)[Other l1li3] is best as the valence-sum rule holds around both cations and anions. However, the inclusion of the anion bond-valence sums in the optimization is quite difficult on a large scale (this issue will be discussed later). Brown & Shannon (1973 ▸) generally used methods (1)[Other l1li1] and (2)[Other l1li2] to derive their parameters. The least-squares optimization was done using the following equation
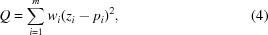
where *Q* is the sum of the residuals, *m* is the number of ions of type *i*, 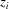 is the valence, 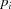 is the bond-valence sum and 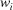 is a weight set to 1/σ^2^(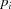), where σ(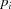) is the standard error on 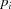. Following the optimization, Brown & Shannon (1973 ▸) evaluated the quality of their parameters by calculating root-mean-square *relative* deviations between the sums of the bond-valences of an ion, and its valence [equation (2)].

#### 9.1.2. Fixing the *B* parameter   

Brown & Altermatt (1985 ▸) proposed a new equation to model the bond-length to bond-valence relation
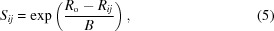
where 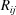 is the bond length between ions *i* and *j*, 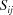 is the bond valence, and *R*_o_ and *B* are the bond-valence parameters. The valence-sum rule requires that
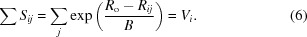
Equation (6) may be rearranged to give equation (7)

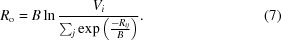
Other than an improved fit, an advantage of this equation is that the *B* parameter adopts a narrow range of values that has a relatively low influence on the resulting bond-valence sums. This led Brown & Altermatt (1985 ▸) to give *B* a fixed value of 0.37 Å for all ion pairs, which allows the exact solution of *R*_o_ for individual cation coordination polyhedra. The value of *R*_o_ for a given ion pair given by Brown & Altermatt (1985 ▸) is the geometric mean value for all cation-coordination polyhedra used in the calculation.

#### 9.1.3. Graphical method: cation and anion sums   

Krivovichev (1999 ▸) pointed out that oxygen ions encapsulated as OPb_4_ clusters consistently show higher-than-expected bond-valence sums at the central anion, and Krivovichev & Brown (2001 ▸) attributed this problem to the choice of bond-valence parameters. They suggested a new method of derivation that refines both *R*_o_ and *B* using the following equation, obtained by rearrangement of the equation of Brown & Altermatt (1985 ▸)
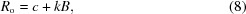
where *c* and 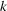 are fitted constants. Equation (8) is refined for both the cations and the anions, and the bond-valence parameters are extracted at the intersection of these curves. Krivovichev (2012 ▸) derived 8 pairs of bond-valence parameters using this method, but pointed out that the introduction of anion-centered coordination polyhedra into the refinement greatly limits the applicability of the method; for structures to be usable, not only must the cation make all bonds to the same anion, but the anion must also make all bonds to that same cation. This constraint is of significant importance in data collection and precludes this method being used for most cation–anion pairs. Furthermore, equation (8) only holds for *B* ≃ 0.30–0.60 Å.

#### 9.1.4. Graphical method: cation sums   

Sidey (2009 ▸) proposed a variation of the method of Krivovichev & Brown (2001 ▸) that also allows simultaneous determination of *R*_o_ and *B* where only the bond-valence sums of the cations are optimized. This enhances the applicability of the approach, and the anion bond valences are checked *a posteriori* to see if they are of acceptable quality. The valence-sum rule [equation (6)] may be rearranged to
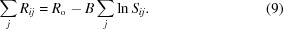
For coordination environments in which all bonds are of the same length, this equation simplifies to
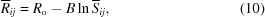
where 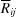 is the mean bond length and 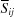 is the mean bond valence.

Graphical representation of 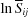 as a function of 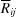 gives *B* as the slope and *R*_o_ as the *y*-intercept from a linear least-squares fit for many coordination polyhedra. However, the constraint of equal bond lengths on the coordination environment greatly restricts the amount of data that can be used with this method. Moreover, the sole use of coordination environments of equal bond length is generally not recommended in the determination of bond-valence parameters, as they cannot appropriately model the relation over the full range of bond lengths of the ion pairs.

Brown (2009 ▸) used an approximation proposed by Urusov (2003 ▸) (in dealing with the distortion theorem) in order to circumvent the constraint on the bonding environment. A Taylor expansion is applied to the mean bond length, 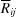, to obtain the adjusted mean bond length, 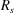


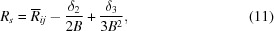
where 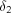 is the mean-square deviation and 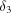 the mean-cube deviation of the bond lengths from the mean bond length 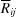. Substituting 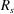 for 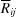 in equation (10) and changing the mean bond-valence 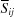 to its ideal value of 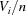, where 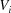 is the valence of the cation and *n* its coordination number:
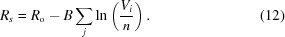
Solution for the bond-valence parameters then follows the same procedure as for equation (10). Although the graphical method is attractive for providing a solution for both *R*_o_ and *B*, and having wide applicability, it suffers a major drawback in addition to the approximation introduced in equation (11): rather than minimizing deviations between the sum of the bond valences and the valence of the ion, the parameters derived by this method are based on minimization of bond-length deviations from the mean, and hence do not relate directly to the valence-sum rule. Moreover, this method assumes that variations in mean bond lengths are solely the result of distortion, whereas variation in coordination number of the anions can also contribute in a major way to variations in mean bond length (*e.g.* Shannon, 1976 ▸). The method also fails for certain ions showing very large gaps in their bond-length distributions (*e.g.* H^+^, Se^4+^, I^5+^, discussed below).

#### 9.1.5. RMSD minimization   

Brown (2002 ▸) proposed minimizing the squared difference between the sum of the bond valences and the valence (oxidation state) of the ion
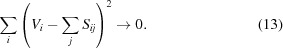
Mills *et al.* (2009 ▸) reformulated this optimization into a minimization of the root-mean-square deviation
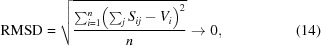
where the minimization is done over *n* cation coordination polyhedra. They generally report their results in v.u. but also sometimes in % deviation per unit of charge [equation (2)]. Whereas equations (13) and (14) lead to the same result, equation (14) is more appropriate for reporting these results, as the squared deviation from equation (13) is extensive, *i.e.* the resultant value is dependent on the number of coordination polyhedra used in the minimization, whereas the RMSD from equation (14) is intensive, *i.e.* it is independent of the number of coordination polyhedra used.

A significant drawback of the RMSD minimization (although not exclusive to it) is found in its weighting scheme. In this minimization, every coordination polyhedron is weighted equally, meaning that the dominant coordination number of a cation can easily dominate the optimization at the expense of others. A classic example of this failure is for Si^4+^, with ∼ 100 times more data for coordination 4 than for coordination 6. When deriving bond-valence parameters for Si^4+^ by minimizing the RMSD, we obtain stellar agreement for coordination number 4, with a mean bond-valence sum (BVS) of 3.99 v.u., and overall (mean BVS 4.00 v.u., RMSD = 0.097 v.u.), but the refined parameters yield a mean bond-valence sum of 4.54 v.u. for coordination 6. We observe this result to various degrees for all ions with multiple coordination numbers, and hence minimizing the RMSD is often not reliable. However, the minimization can be modified to become an integral part of the method of derivation of choice (next).

### 9.2. Generalized reduced gradient (GRG) method of RMSD minimization   

To address the problem described above, we (1) use a weighting scheme that finds a balance between overall fit, and fit on the basis of coordination number, and (2) introduce the use of a new search algorithm.

#### 9.2.1. The generalized reduced gradient method   

The search for the global minimum involving equation (14) has so far been done iteratively, by varying the bond-valence parameters until a minimum, presumably the global minimum, was found (Mills *et al.*, 2009 ▸; Mills & Christy, 2013 ▸). However, this method is not practicable when dealing with more than a handful of ions.

We propose using the generalized reduced gradient (GRG) search algorithm (Abadie & Carpentier, 1969 ▸) in combination with the RMSD minimization. We chose this algorithm because (1) it can deal with the optimization of non-linear equations, (2) it is very efficient (convergence occurs in a matter of seconds), (3) it consistently gives a better fit to the data than other search algorithms used, and always converges to the same value for each ion pair.

**Figure 1:**
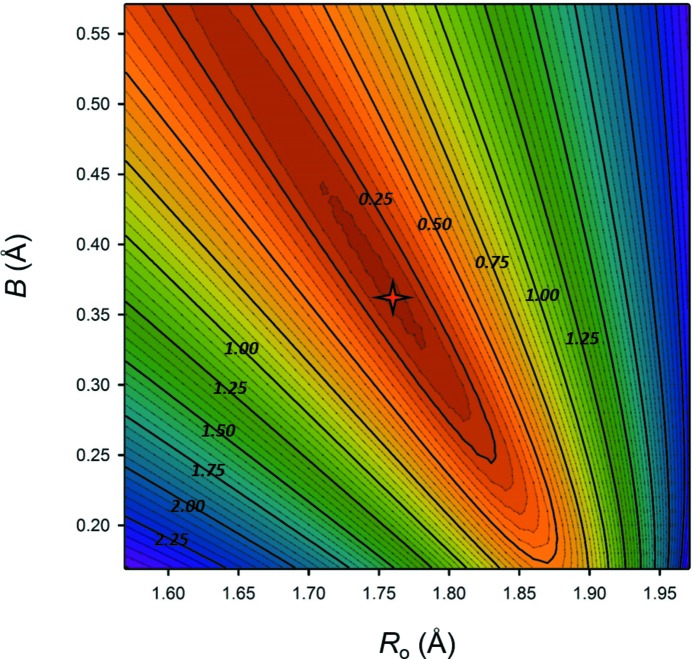
RMSD (v.u.) from the valence-sum rule as a function of the bond-valence parameters for Fe^3+^.

While the GRG optimization has proved to be much more effective than an iterative search method, the use of a search algorithm generally raises concern as to whether the minimization obtained is a local minimum as opposed to the global minimum. Mills & Christy (2013 ▸) show that contour plots of RMSD as a function of *R*_o_ and *B* for Te^4+^ and Te^6+^ are smooth and concave in shape, but the plots only cover a narrow range of values around the extracted parameters. In Fig. 1 ▸ we show (for Fe^3+^) that the shape remains concave over a much larger range of values, and no maxima, saddle points or other minima are observed. As a result, convergence can only lead to the global minimum. Note that both Fig. 1 ▸ and the plot of Mills & Christy (2013 ▸) show that the contour lines can have a pronounced oval shape; thus different combinations of values for *R*_o_ and *B* can lead to the same level of fit over a non-negligible range of values for the cations (although different parameters from one contour line may give different anion BVS), which may be deceptive in an iterative search for the global minimum.

#### 9.2.2. Weighting scheme   

To deal with the weighting issue for the different coordinations of an ion (as discussed for Si^4+^), we introduced a second optimization criterion where we additionally minimize the RMSD between the mean bond-valence sum of the observed coordination numbers of an ion and the oxidation state of that ion (*i.e.* coordination-based RMSD minimization). Following experimentation with weighting schemes, we concluded that a 2:1 ratio between overall RMSD and coordination-based RMSD gave the best results, in keeping the overall RMSD low while supressing the dominant effect of certain coordination numbers.

Hence, the GRG method of RMSD minimization proposed here implicitly entails optimization on both the overall and coordination-based RMSD (denoted hereon as the GRG method), and addresses many shortcomings of the other methods of derivation in that it (1) refines both bond-valence parameters, (2) optimizes the appropriate quantity, (3) does not require approximations and (4) is universally applicable. The main drawback of this new method (although it is a drawback of most methods) is that it does not optimize the anion bond-valence sums. However, as will be discussed below, optimizing the anion bond-valence sums may not be necessary, and is not practical on the scale of this study. Where using this method, the bond-valence sums of the anions are tested *a posteriori*.

In this work, the GRG method used a multi-start approach of 1000 random starting pairs of variables until convergence to the fourth decimal place using forward derivative.

### 9.3. Comparison of the most common methods of derivation   

First we will focus on ions that occur in more than one coordination by O^2−^; of the 135 ions examined here, 45 have only one coordination number and 90 have more than one coordination number. Table 1 ▸ compares two common methods of derivation to the GRG method for the 90 ions. The first and second columns give the ion identity and number of coordination polyhedra obtained in our bond-length dispersion analysis, respectively. The third column gives the RMSD of the set of best published parameters, taken from Table S1. The fourth column gives the RMSD obtained using the graphical method [equation (12)]. The fifth column gives the RMSD by setting *B* = 0.370 Å and refining *R*_o_ in the same way as the GRG method, and the last column gives the RMSD values for the GRG method, refining both *R*_o_ and *B*.

We use the set of best published parameters (Table S1) as a benchmark to evaluate the other methods. Table 1 ▸ shows that the graphical method gives better parameters for 59 of the 90 ions with more than one coordination number, 62 for the method of fixing *B* at 0.37 Å, and 76 for the GRG method. One of the major problems of the graphical method is observed for ions showing large gaps in their bond-length distributions, such as Se^4+^ (RMSD = 47.223 v.u.), Te^4+^ (4 × 10^4^ v.u.), I^5+^ (108.803 v.u.), where the approximation of the ‘adjusted mean bond length (*R*_s_)’ fails, and unusual values of the bond-valence parameters are obtained (*e.g. R*_o_ = 2.119 and *B* = −0.052 for Te^4+^).

In terms of the mean weighted-RMSD (weighted by the number of coordination polyhedra), a higher value is obtained for the graphical method than for the set of best published parameters with values of 0.161 v.u. (7.96% per unit of charge) and 0.140 v.u. (6.5% per unit of charge), respectively, despite omitting the ions with RMSD > 1 v.u. in the calculation for the graphical method. The method of fixing *B* gives an overall fit similar to the set of best published parameters, with a mean weighted-RMSD of 0.139 v.u. (6.7% per unit of charge). In contrast, the GRG method shows significant lowering of the mean weighted-RMSD with 0.128 v.u (6.1% per unit of charge).

These results for the GRG method are welcome improvements, and confirm our choice of a 2:1 weighting scheme between overall RMSD and coordination-based RMSD. This weighting scheme thus allows a significant improvement in overall fit for cations, without sacrificing the fit of the different coordination numbers of the cations (*i.e.* allowing a very small increase in the RMSD leads to overwhelmingly better agreements over the entire range of coordination numbers of an ion). Moreover, we found the GRG method to give much better bond-valence sums for the anions than a regular RMSD minimization (see below).

### 9.4. General considerations   

#### 9.4.1. Optimizing both cation and anion bond-valence sums   

The valence-sum rule states that the sum of the bond valences for an ion is equal to the valence of that ion (Brown, 2002 ▸), and does not discriminate between cations and anions. The tendency to focus on the bond-valence sums of the cations more than those of the anions arises from the fact that cation-centered coordination polyhedra commonly involve a single type of anion, whereas anion-centered coordination polyhedra commonly do not, although exceptions such as MgO and NaCl do occur.

Modifying equation (14) to include the 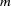 anions in the summation leads to
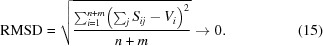



Equation (15) can be solved in two ways to extract the bond-valence parameters. First, the optimization can be done on the basis of individual crystal structures, and the resulting parameters are averaged over all crystal structures used. However, the requirement of having at least two unique and linearly independent coordination environments (*e.g.* two cation environments in different coordination numbers, or one cation environment and one anion environment for the same ion pair) for every bonded pair of ions in every crystal structure renders this method practically inoperable.

The second (and more conventional) way of solving equation (15) consists of optimizing the bond-valence sums on the basis of single coordination polyhedra. In this case, bonding environments are recorded for both cations and anions. A single optimization is then run for the coordination polyhedra of all crystal structures combined, to simultaneously solve for the bond-valence parameters of all pairs of ions. Whereas this removes the constraint on the bonding environments that makes the solution on the basis of individual crystal structures impractical, this method introduces a new drawback: the ensuing optimization results in a large system of (non-linear) equations, of dimension *D* where
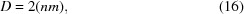
where 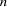 and *m* are the numbers of cations and anions, respectively, and *D* is the minimum number of linearly independent equations required to solve equation (15) for all bond-valence parameters. Furthermore, any sensible attempt at solving equation (15) using this approach necessarily entails a highly over-determined system of equations for the results to be significant. In this study, we have 135 cations bonded to a single anion (O^2−^), and thus 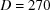. Whereas we need a minimum of 270 equations to solve equation (15) for the 135 pairs of bond-valence parameters, we derived 31 521 non-linear equations from the valence-sum rule, for the cation coordination polyhedra alone. We did not collect the bond-length data of anion-centered coordination polyhedra, but the number of resulting equations would be somewhat similar. While the simultaneous optimization of ∼ 60 000 270-dimensional non-linear equations may not be impossible, this kind of calculation is very impractical.

A number of approximations can be made to circumvent this problem: (1) limit the number of ion environments in the refinement to only a couple of ions (*e.g.* Krivovichev, 2012 ▸), (2) increase the ‘universality’ of the parameterization to lower the number of bond-valence parameters (*e.g.* one pair per isoelectronic series; Brown & Shannon, 1973 ▸), or (3) optimize the cation bond-valence sums only, and verify that the anion sums work *a posteriori*.

#### 9.4.2. On the universality of the bond-valence equation   

The universality of the bond-length to bond-valence relation is generally understood to mean the transferability of the relation between pairs of ions from structure to structure. However, it is important to realise that selection of the level of universality on the basis of pairs of ions is arbitrary, and depends on the quality of fit desired.

Bond-valence parameters can be derived with different levels of universality. Coulomb’s law, which is arguably at the core of the relation (Preiser *et al.*, 1999 ▸), offers an extreme case where only eight pairs of bond-valence parameters are required to model all ions bonding to oxygen (*i.e.* a pair of bond-valence parameters for each cation oxidation state, 1+ to 8+). However, this parameterization would yield a very poor fit due to structural and electronic effects that are not transferable between ions of the same charge. Conversely, reducing the universality from ion pairs to (for example) specific coordination environments could increase the fit to the valence-sum rule slightly, although at the cost of a more cumbersome parametrization. Two levels of universality are compared by Brown & Shannon (1973 ▸) in their initial description of the relation. They derive parameters based on isoelectronic series, reporting a root-mean-square *relative* deviation [equation (2)] of 5.4% per unit of charge for a total of 27 ion pairs, compared with 4.0% per unit of charge for parameters derived for individual ion pairs. This result led to a widespread use and derivation of parameters based on ion pairs, which today still seems like the best compromise between universality and fit.

#### 9.4.3. Minimization using *a priori* bond valences   

*A priori* bond valences (called *theoretical* by Brown, 1987 ▸; and *ideal* by Brown, 2013 ▸) are obtained by solution of the network equations of a crystal structure (see Brown, 2002 ▸). Brown (2002 ▸) suggests optimizing the bond-valence parameters on the basis of minimization of the squared difference between observed and *a priori* bond valences. This approach has not yet been examined, and we note that it is not equivalent to the methods examined above (§9.1). Aside from the method of Krivovichev (Krivovichev & Brown, 2001 ▸; Krivovichev, 2012 ▸), the methods examined above rely on minimizing the RMSD of the bond-valence sums around the cations and omit consideration of the anions. As *a priori* bond valences are derived from all the valence-sum-rule equations in a structure (usually augmented by loop equations), it follows that optimizing bond-valence parameters with reference to observed and *a priori* bond valences is equivalent to optimizing the valence-sum rule for all ions (cations and anions) in a structure. However, this method faces a similar constraint as for the solution of equation (15): a very large number of high-dimensional non-linear equations to solve.

### 9.5. The bond-length–bond-valence equation   

We now have an effective method for the derivation of bond-valence parameters (the GRG method), and have determined that (1) the minimization should be done on the cation bond-valence sums, while the anion bond-valence sums are verified *a posteriori*, and (2) the most useful level of universality remains on the basis of ion pairs. In this section, we use these criteria to examine new potential equations to describe the bond-length–bond-valence relation.

#### 9.5.1. Evolution of the bond-length–bond-valence equation   

Generalization of equation (14) shows that the desired optimization entails minimization of the difference between the valence of the cation *V_i_* and some function of the independent parameter 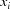, 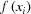


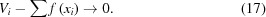
Pauling (1929 ▸) first used coordination number as the independent parameter
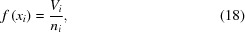
where 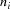 is the coordination number of cation *i*. Taking the sum on each side of equation (18)

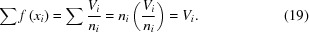



Equation (17) [and by extension equation (14)] has an exact solution
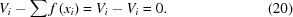
In short, Pauling suggested an exact solution to equation (14). However, with this parameterization, there is the lack of correspondence between the resulting anion bond-strength sums and the oxidation states of the anion(s).

Pauling (1947 ▸) proposed using bond length as a parameter in describing electron-sharing in metallic bonds
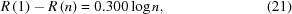
where *R*(1) is the length of the shortest bond in the coordination polyhedron, *R*(*n*) is the length of the bond considered, and *n* is the bond number (the number of bonding electrons). This equation was used by Byström *et al.* (1951 ▸) to show (from bond-length considerations) that the sum of the bond numbers around V in V_2_O_5_ is 4.96, fairly close to the vanadium oxidation state of 5. Subsequent contributions to the relation (Smith, 1953 ▸; Zachariasen, 1954 ▸, 1963 ▸; Zachariasen & Plettinger, 1959 ▸; Evans & Mrose, 1960 ▸; Evans, 1960 ▸; Pant & Cruickshank, 1967 ▸; Clark *et al.*, 1969 ▸; Perloff, 1970 ▸, Donnay & Allmann, 1970 ▸) led to a major advance in the parameterization of 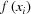 by Brown & Shannon (1973 ▸) who proposed a *universal* correlation between bond length and bond strength (transferable from structure to structure) using equation (2). This equation was later updated by Brown & Altermatt (1985 ▸) to equation (5), which is still in use today. Other equations have been proposed (Ziółkowski, 1985 ▸; Naskar *et al.*, 1997 ▸; Valach, 1999 ▸; Mohri, 2000 ▸) that commonly attempt to give a physical justification to the bond-length–bond-valence relation, but they are of more complicated form and have not seen wide application.

Note that the choice of bond length as a parameter, although well ingrained in the bond-valence method, is not required by bond-valence *theory*, as none of the three axioms of the theory (see Brown, 2002 ▸) mention bond lengths, and it is feasible in principle that other parameters could be found in the future.

#### 9.5.2. Derivation of new equations   

In deriving new equations to model the relation, we must keep in mind that the number of coordination environments required to solve for the bond-valence parameters of a pair of ions is at least equal to the number of parameters of the equation used in describing the relation. In other words, the addition of parameters to increase the degree of fit is not without consequences, as many ions have few different coordination numbers. The conventional choice of a two-parameter equation to represent the bond-valence relation means that at least two distinct coordination environments are necessary to solve for the parameters of the equation. As noted above, of the 135 ions examined in this work, 45 ions occur in only one coordination, making this a common and significant problem in the derivation of bond-valence parameters.

To derive new equations for the description of the bond-length to bond-valence relation, we focused on a specific ion assumed to be representative of the relation. We used Al^3+^ for this purpose as (1) it covers a wide range of bond valences, and (2) a large amount of structural data on crystals containing Al^3+^ is available.

51 crystal structures containing Al^3+^ were selected from the Inorganic Crystal Structure Database (ICSD), following a strict set of filtering criteria: (1) the site of interest is fully occupied by Al^3+^; (2) *R*_1_ < 0.03; (3) the structure contains no H; (4) all sites in the structure are fully occupied and show no positional disorder; (5) the structure is not extensively strained; (6) the structure contains no ions showing known stereochemical electronic effects (*e.g.* ^[6]^Cu^2+^, ^[6]^Mn^3+^); (7) crystallographic data were measured at ambient conditions; (8) there is no heterovalent solid solution at any site; (9) there is no more than 10% homovalent solid-solution at any site other than that occupied by Al^3+^. The coordination polyhedra must also be clearly defined; any doubt resulted in a discarded entry. Table S2 gives the ICSD code of the resulting 51 crystal structures, their *R* value (mean = 0.019), and the number of Al-centered coordination polyhedra used for each structure (for a total of 90). Note that duplicate structure types are used, as long as there is a significant change in site occupancy.

**Figure 2:**
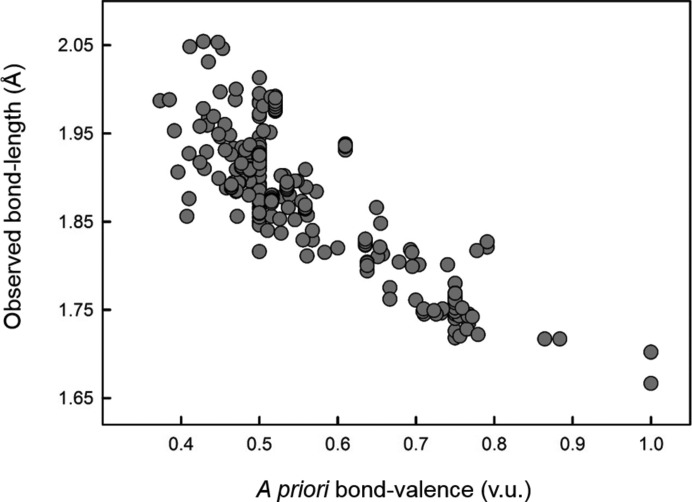
Determination of the curvature of the bond-valence relation by a match of the *a priori* bond valences to their observed bond lengths.

The network equations were derived for each of the 51 crystal structures to determine their *a priori* bond valences using the method of Rutherford (1990 ▸). The *a priori* bond valences were then compared with their respective experimental bond lengths, for a total of 481 pairs. The resulting plot is shown in Fig. 2 ▸. A series of simple equations were then fitted to the data points by least-squares minimization. These equations are considered next.

#### 9.5.3. Two-parameter equations: sample evaluation   

The top 17 two-parameter equations obtained in the above fitting procedure were selected for further analysis. These equations are given in Table 2 ▸ and are identified by the numbers in square brackets. They include the exponential equation of Brown & Altermatt (1985 ▸) as equation [1]. We group the three equations containing an external parameter (a parameter that is not a multiplier of *x*) as equations [15]–[17], and add two more equations: the original equation of Brown & Shannon (1973 ▸), equation [18], and an expression related to the Born–Landé (1918 ▸) equation, equation [19].

To evaluate these equations, we considered eight relatively common ions that cover different types of bonding behavior: Na^+^, Al^3+^, Si^4+^, Ca^2+^, Mn^2+^, Mo^6+^, La^3+^ and Pb^2+^. We used the GRG method to derive bond-valence parameters for each of these eight ions bonded to O^2−^ for the 19 equations of Table 2 ▸.

Table 2 ▸ gives the RMSD obtained by the GRG method for each equation, for each ion. Thus, the current form of the relation, equation [1], gives a mean RMSD of 0.132 v.u for the ions considered. Many equations give a RMSD similar to that of equation [1], and five of the 19 equations ([3], [4], [11], [14] and [19]) give an equal or slightly lower mean RMSD (including the expression related to the Born–Landé equation). The original equation of Brown & Shannon (1973 ▸), equation [18], also gives reasonable results with a mean RMSD of 0.134 v.u. for the sample of ions considered. The mean RMSD of the 14 best-fit equations with no external parameters ([1]–[14]) is 0.148 v.u. The top three equations with one external parameter (equations [15]–[17]) have a mean RMSD of 0.144 v.u., which indicates that although the presence of an external parameter removes some flexibility in the shape of the curve, it does not necessarily reduce the fit.

#### 9.5.4. Two-parameter equations: full evaluation of best equations   

Next, we selected six two-parameter equations that gave a similar or better fit to that of the equation of Brown & Altermatt (1985 ▸; equation [1]) on the sample of eight ions (equations [2], [3], [4], [14], [15], [19]) and compared them to that equation for the 90 multiple-coordination-number ions of our bond-length dispersion analysis. Bond-valence parameters were derived for the seven equations, for each of the 90 ions, using the GRG method of derivation. The resulting RMSD for the six equations obtained for the 90 ions are given in Table S3. Using the number of coordination polyhedra for each ion as a weighting factor, equations [3], [4] and [14] give a mean weighted-RMSD of 0.128 v.u., equations [2] and [19] 0.129 v.u., and equation [15] 0.13 v.u., compared with 0.128 v.u. (Table 1 ▸) for equation [1].

In addition to the similarity of the overall values, there is little spread in the RMSD values of the different equations of Table S3 (mean standard deviation of 0.005 v.u. on the basis of ions). This leads to two conclusions: (1) many equations (and in various forms) can describe the relation, and (2) we have likely reached a plateau in the fit for two-parameter equations. It is notable that the equation of Brown & Altermatt (1985 ▸) leads to the best fit (tied here with equations [3], [4] and [14]), even though we derive the equation and its bond-valence parameters in a different way than Brown & Altermatt, and on a very different dataset. This is a welcome result, as it does not warrant update of the well established ‘exponential equation’; only an improved set of bond-valence parameters is needed.

#### 9.5.5. Three-parameter equations: sample evaluation   

Next we examined three-parameter equations using the same procedure as above. Six three-parameter equations were selected for evaluation over the sample of ions (above) and are given in Table 3 ▸ (equations [20]–[25]). We also add a third (external) parameter to the best two-parameter equation of Table 2 ▸ (equation [26]), bringing the total to seven three-parameter equations.

The lowest mean weighted-RMSD for the sample was obtained for equations [20]–[22] with a value of 0.119 v.u., compared with 0.130 v.u. for the best two-parameter equations (Table 2 ▸, equations [3] and [14]). Despite the decrease in RMSD, there are three drawbacks that make the three-parameter equations less attractive: (1) the derivation of the bond-valence parameters requires at least three coordination environments per ion; (2) the search for the global minimum of the RMSD (for evaluation of the bond-valence parameters) becomes much less reliable as the RSMD landscape (*i.e.* Fig. 1 ▸) becomes more complicated; (3) the bond-valence parameters cannot be interpolated to ions with less than three coordination numbers because of high variability, and, of the 135 ions used here, 64 ions have less than three coordination numbers. Thus, three-parameter fits do not seem desirable, at least at the present time.

#### 9.5.6. The bond-valence equation: conclusions   

From our search for a new equation, we conclude that (1) many equations can model the bond-length–bond-valence relation adequately; (2) the loss of trends in the bond-valence parameters for three-parameter equations discourages their use; (3) the current form of the relation given by Brown & Altermatt (1985 ▸) shows the best compromise between applicability and fit.

### 9.6. New bond-valence parameters   

New bond-valence parameters were derived in the same way as for the trial equations (above), that is by minimization of the difference between the sum of the bond valences of an ion and its valence using the GRG method. The bond-valence parameters of the 90 ions with more than one coordination number are given in Table 4 ▸ for the equation of Brown & Altermatt (1985 ▸). Table 4 ▸ also includes 45 additional pairs of parameters for those ions with only one coordination number, to bring the total to 135 pairs of bond-valence parameters. The parameters for the 45 additional ions are identified by a number (from 1 to 3), depending on how these parameters were derived (see below).

#### 9.6.1. Trends in the bond-valence parameters for ions with two or more coordination numbers   

**Figure 3:**
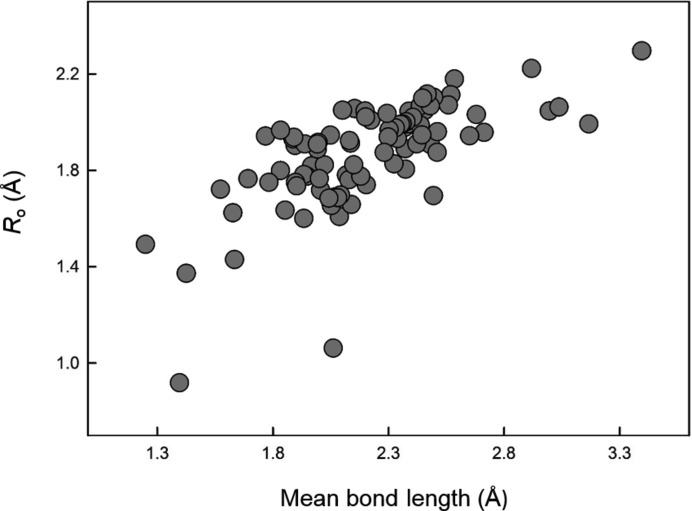
Bond-valence parameter *R*_o_ as a function of mean bond length for the 90 multiple-coordination-number ions

Here we will examine trends in the bond-valence parameters derived with the GRG method for the 90 ions with two or more coordination numbers. We begin with the relation between the bond-valence parameter *R*_o_ and the mean bond length of a pair of ions. Fig. 3 ▸ shows our new values for *R*_o_ as a function of mean bond length. The correlation is not strong (*R* ^2^ = 0.457), although it changes slightly by removing the two lower outliers (H^+^, Li^+^; *R* ^2^ = 0.516). Certain groups of ions of similar crystal-chemical behavior also show significant correlation (*e.g.* alkali metals, *R* ^2^ = 0.937; alkaline-earth metals, *R* ^2^ = 0.962). Attempts to relate the individual parameters *R*_o_ and *B* directly to other physical properties of the ions were not successful.

**Figure 4:**
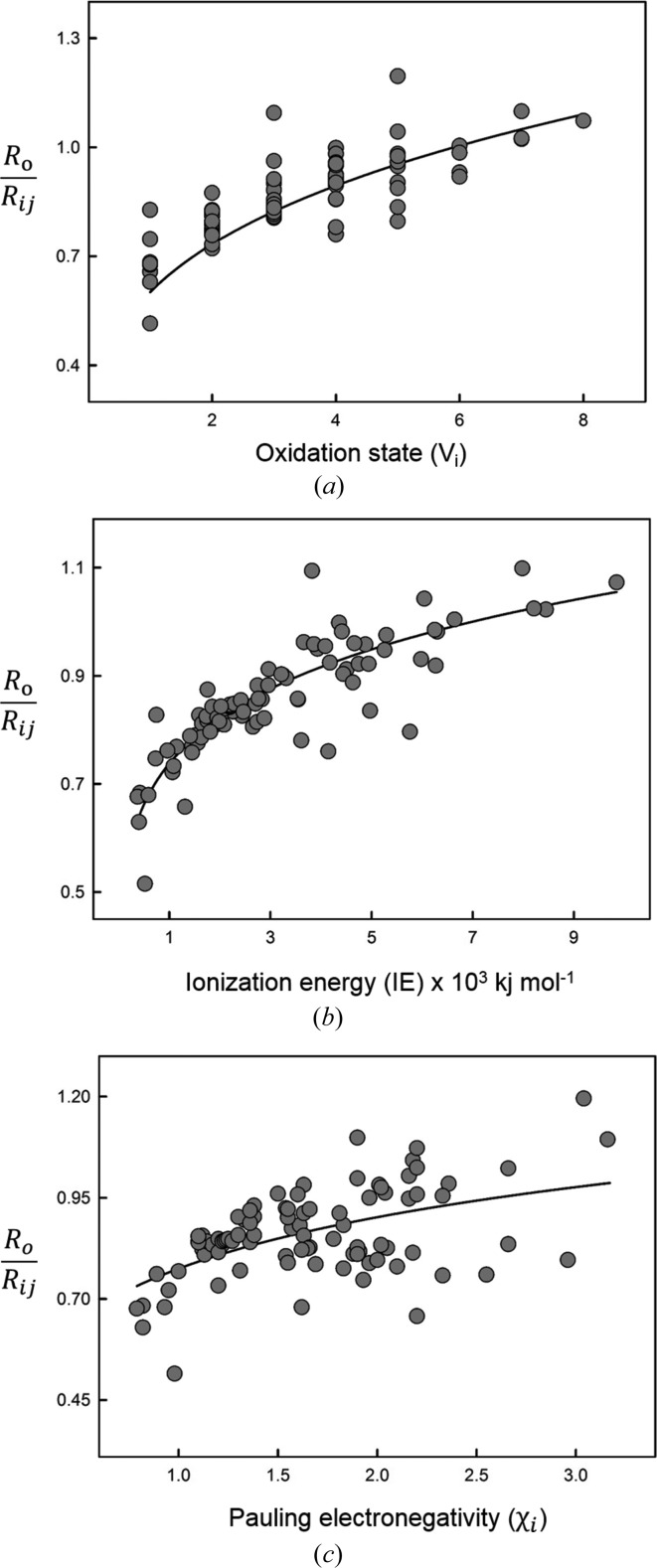
Relation between bond-valence parameter *R*_o_ divided by mean bond length and (*a*) oxidation state, (*b*) ionization energy and (*c*) Pauling electronegativity.

On the other hand, the ratio *R*_o_/〈*R_ij_*〉 shows significant correlation with various cation properties: (1) oxidation state, *V_i_*; (2) ionization energy, *IE*; and to a much lesser extent (3) Pauling electronegativity, 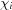


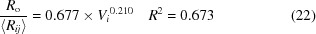


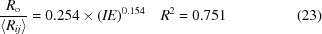


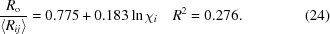
These relations are shown in Fig. 4 ▸. We use the Pauling electronegativity scale (Pauling, 1960 ▸) as it gives a slightly better value for *R* ^2^ (0.276) compared with the scales of Allen (Allen, 1989 ▸; 0.272) and Allred–Rochow (Allred & Rochow, 1958 ▸; 0.262). Similarly, Brese & O’Keeffe (1991 ▸) derived a correlation between *R*_o_ and a combination of (Allred–Rochow) electronegativity and an empirically derived ‘size parameter’. To evaluate the reliability of equations (22)–(24) [Disp-formula fd23] [Disp-formula fd24], we calculate the mean absolute deviation between the values of *R*_o_ predicted by these equations, and those derived by the GRG method for all usable ions. Equations (22)–(24) [Disp-formula fd23] [Disp-formula fd24] give mean deviations of 4.89, 4.21 and 9.00%, respectively. Even though the deviations calculated from equations (22) and (23) seem reasonable, one must be careful when using these equations to interpolate values for *R*_o_. Thus, for equation (23) the experimental value of *R*_o_ falls within the range of its predicted value with error for only 61 of the 90 ions. Moreover, deviations on *R*_o_ have a much larger effect on the bond-valence sums than deviations on *B*. As a result, it is much safer to fix *B* to a reasonable value (such as the mean value of 0.399 Å) rather than fixing *R*_o_, when dealing with uncommon cations observed in only one coordination.

#### 9.6.2. The one-coordination-number problem   

As noted above, the choice of a two-parameter equation to represent the bond-valence relation [equation (5)] means that at least two distinct coordination environments are required to solve for the parameters of the equation. Of the 135 ions examined here, 45 ions occur in only one coordination, resulting in a significant problem with regard to the calculation of their bond-valence parameters. Several ways around this ‘one-coordination-number problem’ have been proposed. For example, Brown & Shannon (1973 ▸) used the bond-length information for the same cation in different coordination and bonded to other anions, and adjusted the bond lengths in proportion to the difference in ionic radius of the anions, while Brown & Altermatt (1985 ▸) fixed the *B* parameter to 0.37 Å, which effectively removes the factor of 2 in equation (21). Other ways of dealing with this problem (*e.g.* Krivovichev & Brown, 2001 ▸) are applicable only to a small set of data.

#### 9.6.3. Interpolation to ions with only one coordination number   

Here, we explore different options for fixing one of the bond-valence parameters for the 45 ions with only one coordination number, using the trends in the bond-valence parameters described above. We use three different methods that involve fixing either *R*_o_ or *B*, and letting the other parameter refine by the GRG method.

Although the system is underdetermined, any useful solution must be physically realistic and consistent with the results obtained for the ions observed in multiple coordination numbers. Thus, we have an idea of the range that calculated values of *R*_o_, *B* and of the RMSD should occur within, based on ions showing similar crystal-chemical behavior as well as for all ions considered. We experimented with fixing both *R*_o_ and *B*, and found that ions should be treated on a case-by-case basis. The following three methods of derivation were used to derive the bond-valence parameters of ions observed in only one coordination:

(1) Fix *R*_o_ to the value predicted by equation (22) (ionization energy), or to the mean of the values predicted by equations (22) and (23). Let *B* refine, and see if the values for both *B* and the RMSD fall within a reasonable range for that family or group of ions with similar crystal-chemical behavior. If this is not the case, move to method (2)[Other l2li2].
(2) Fix *B* to a reasonable value based on family (*e.g.* for the transition metals, the mean value of *B* is 0.375 Å) or group of ions with similar crystal-chemical behavior. Let *R*_o_ refine, and see if both *R*_o_ and the RMSD fall within a reasonable range for that family. If this is not the case, move to method (3)[Other l2li3].
(3) Fix *B* to the mean value for all multiple-coordination-number ions combined (0.399 Å) and let *R*_o_ refine. This is done where (1)[Other l2li1] and (2)[Other l2li2] fail, or where there is insufficient data available to make a reasonable estimate of *B* (*e.g.* for the non-metals).

For the 45 ions considered, we fixed *R*_o_ for 22 ions and *B* for 23 ions. Parameters derived by fixing a parameter are identified by their method of derivation (1, 2 or 3) in Table 4 ▸.

### 9.7. Precision   

Several factors affect the precision of the RMSD and bond-valence parameters calculated in this work: (1) uncertainty in the experimental bond lengths, (2) uncertainty in the parameterization of the model, and (3) the presence of structural strain in the bond-length data. We estimated the effect of (1) by taking an average standard deviation for a bond length (∼ 0.005 Å) and, using a sample of nine ions (H^+^, Na^+^, Mg^2+^, Al^3+^, S^6+^, Zn^2+^, La^3+^, Pb^2+^, Th^4+^), determined the effect of varying bond lengths by ± 0.005 Å on the RMSD. The uncertainty on the bond length resulted in variations in the third decimal of the RMSD (first decimal for the relative RMSD). The error on *R*_o_ and *B* was then determined by incrementally varying the value of these parameters until the same variation in the RMSD was observed. The uncertainty on both *R*_o_ and *B* caused by (1) is thus determined to be in the third decimal place. On the other hand, while we strived to minimize the effect of uncertainty in the parameterization of the model in this work (2), simple factors such as the largely variable sample sizes of the ions affect the accuracy and precision of the results in ways that are arguably more important than the uncertainty on the experimental bond lengths. As for the presence of structural strain (3), this phenomenon is structure dependent and cannot (currently) be evaluated by any method not dependent on the valence-sum rule. We thus give the values to be precise to the third decimal as a best-case scenario.

## 10. Anion-sum verification   

Bond-valence parameters are usually derived on the basis of the cation bond-valence sums. However, as discussed earlier, parameters are expected to work equally well for both cations and anions according to the valence-sum rule. If a method of derivation is selected that optimizes the bond-valence sums for cations only, it is critical that these parameters be evaluated *a posteriori* to check that they also work well for anions. As this is seldom done, here we will evaluate the anion bond-valence sums for four sets of parameters: (1) those of Brown & Altermatt (1985 ▸), (2) those of Brese & O’Keeffe (1991 ▸), (3) the set of best published parameters from Table S1, and (4) the new parameters given in this work.

We assembled a set of structures covering all pairs of bond-valence parameters derived in this paper (with at least one unique structure per cation), unless no structure could be evaluated solely with the parameters of our dataset. This resulted in a set of 128 structures (Table S4). The structures were then evaluated with the three (smaller) sets of parameters given above, where applicable, which resulted in four overlapping sets of evaluated structures shown in Table 5 ▸ (note that the way the structure sets are assembled, less common cations become part of the evaluation as the sets get larger.). We used the program *KDist* (part of the Kalvados software suite; http://www.fzu.cz/~knizek/kalvados) to calculate the overall RMSD of the anion bond-valence sums for the different sets of parameters over the structure sets.

The results are summarized in Table 5 ▸ together with the number of structures and the number of anion-centered coordination polyhedra used for each set. For the set of 72 structures, the set of best published parameters as well as the parameters given in this work give the best anion bond-valence sums, with an overall RMSD of 0.100 v.u. (5.0% per unit of charge), compared with 0.119 and 0.130 v.u. for the sets of Brese & O’Keeffe (1991 ▸) and Brown & Altermatt (1985 ▸), respectively. Over the set of 100 structures, the parameters of this work give the best anion bond-valence sums, with a RMSD of 0.099 (4.9% per unit of charge), compared with 0.106 and 0.121 v.u. for the set of best published parameters and the parameters of Brese and O’Keeffe, respectively. The structure set covering 122 unique ions shows even greater distinction between the parameters of this work and what we identified to be the set of best published parameters, with an overall RMSD of 0.103 v.u. (5.1% per unit of charge) compared with 0.120 v.u. Finally, the set of structures covering 128 unique ions yields an overall RMSD of 0.104 v.u. (5.2% per unit of charge) for the parameters derived here.

**Figure 5:**
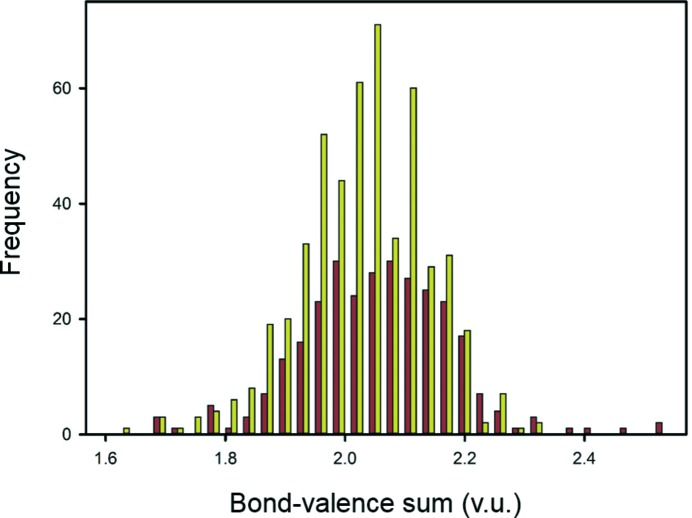
Anion bond-valence sums for the parameters of Brown & Altermatt (1985 ▸; dark red) and the parameters given in this paper (yellow), with sample sizes of 296 and 511 anion coordination polyhedra, respectively.

Fig. 5 ▸ shows the bond-valence sums for O^2−^ for the parameters of Brown & Altermatt (1985 ▸) and the parameters given in this paper. Although the parameters given in this paper account for more coordination polyhedra (511 *versus* 296), the range of bond-valence sums is smaller (1.63–2.30) compared with that obtained from the parameters of Brown & Altermatt (1.67–2.52). The mean bond-valence sum for the parameters of this paper is 2.02 v.u. compared with 2.04 v.u. for the parameters of Brown & Altermatt, with standard deviations of 0.10 and 0.12 v.u. respectively.

We conclude that the parameters given in this paper give the best anion bond-valence sums of the large sets of parameters, in addition to giving the best bond-valence sums for the cations (above). Moreover, the results show that the approximation of deriving bond-valence parameters on the basis of cation coordination polyhedra is justified.

## 11. Improvement in fit: cations   

It is more difficult to compare different sets of bond-valence parameters in terms of cation bond-valence sums, as the sets of parameters often cover a wide array of cations that have different expected levels of fit. However, we can safely compare the two largest sets of parameters discussed above, which are those given in this paper and the set of best published parameters from Table S1.

The parameters given in this paper yield a mean weighted-RMSD of 0.128 v.u. (6.1% per unit of charge) over 31 515 coordination polyhedra for 129 of the 135 ions (9 cations are only found in only one coordination polyhedron, for which the RMSD calculation is irrelevant). On the other hand, the set of best published parameters gives a mean weighted-RMSD of 0.136 v.u. (5.7% per unit of charge) over 31 489 coordination polyhedra for 128 ions. To put things in perspective, the mean weighted-RMSD for the anion bond-valence sums using the parameters of this paper (0.104 v.u.) is 5.2% per unit of charge, slightly lower than for the cation bond-valence sums, although this may be the result of a much smaller sample size.

We usually observe small but consistent improvements in the overall RMSD for most ions in comparison to the set of best published parameters. Where the overall RMSD is not improved (*e.g.* Be^2+^), this is usually because the GRG method gave parameters with a slightly higher overall RMSD in order to compensate for the coordination-based RMSD. The bond-valence sums for coordination numbers 3 and 4 for the best published parameters for Be^2+^ are thus 1.901 and 2.010 v.u., whereas they are 2.000 and 2.000 v.u for the parameters of this work. Major improvements are generally associated with less common ions and are presumably the result of higher quality and/or of more data now being available. For example: for some less-common transition metals, the RMSD for Os^8+^ changes from 0.608 to 0.233 v.u., and Re^7+^ from 0.923 to 0.191 v.u.; for some actinide ions, Np^5+^ changes from 0.820 to 0.061 v.u., and Np^6+^ changes from 1.209 to 0.083 v.u.

The RMSD for the hydrogen ion improved slightly from 0.035 v.u. (for the parameters of Grabowski, 2000 ▸) to 0.033 v.u., which reaffirms that one pair of bond-valence parameters is sufficient to model the ion.

## 12. Deviations from the valence-sum rule   

Of the 462 configurations of ions and coordination numbers examined here, 55 have overall mean bond-valence sums that deviate from the valence-sum rule by more than 0.1 v.u., 11 by more than 0.2 v.u. and 2 by more than 0.3 v.u. In terms of relative deviation from the valence-sum rule, 57 configurations have overall mean bond-valence sums that deviate by more than 2% per unit of charge, 12 by more than 5%, and 2 by more than 10%. The larger deviations are usually unavoidable by using any set of parameters, and are usually for (1) the low/high coordinations of ions observed in many different coordination numbers (*e.g.* Tl^+^, alkali metal ions), (2) structure refinements of dubious quality, and (3) configurations with very little data. The deviations are significantly higher when using other sets of parameters, and show that the addition of the coordination-based RMSD minimization of the GRG method of derivation is valuable.

## 13. Summary   

(1) Evaluation of 244 pairs of bond-valence parameters for 128 cations bonded to oxygen shows a wide variation in the quality of fit to the valence-sum rule, based on 180 194 (filtered) bond lengths from 31 489 coordination polyhedra from 9367 crystal-structure refinements.

(2) We have evaluated two common methods for the derivation of bond-valence parameters: (1) the graphical method, and (2) fixing *B* and solving for *R*_o_. We conclude that both (1) fixing *B* at 0.37 Å, and (2) fixing *B* and solving for *R*_o_. We conclude that both (1) and (2) are not ideal, and we introduce a new method of derivation, the GRG (Generalized Reduced Gradient) method, that leads to better agreement with the valence-sum rule for both cation and anion bond-valence sums.

(3) We have evaluated 19 two-parameter equations and 7 three-parameter equations to model the bond-valence–bond-length relation. We conclude that (1) several equations can describe the relation to a similar degree of accuracy; (2) we have likely reached a plateau in the degree of fit for two-parameter equations; and (3) the equation of Brown & Altermatt (1985 ▸) is best on the basis of fit and practicality.

(4) We have derived new bond-valence parameters for 135 cations bonded to O^2−^ using the GRG method. These parameters give better bond-valence sums for the cations, with a mean weighted-RMSD of 0.128 v.u. (6.1% per unit of charge) for 129 ions and 31 515 cation coordination polyhedra, compared with 0.136 v.u. (5.7% per unit of charge) for what we have determined to be the set of best published parameters over 128 ions and 31 489 coordination polyhedra.

(5) The parameter *R*_o_/〈*R_ij_*〉 is correlated with ion valence (*R* ^2^ = 0.673) and ionization energy (*R* ^2^ = 0.751), indicating that the potential correlation between *R*_o_ and *B* does not adversely affect the derivation of bond-valence parameters provided an effective method of derivation is used.

(6) There are small but consistent improvements in the overall RMSD for most ions in comparison to the set of best published parameters, and excellent improvements in the coordination-based agreement between bond-valence sum and oxidation state. Moreover, some ions show a striking improvement in fit compared with published parameters, likely due to the availability of higher quality data: for example, Os^8+^ changes from 0.608 to 0.233 v.u., Re^7+^ from 1.000 to 0.276 v.u. and Np^6+^ from 1.209 to 0.078 v.u.

(7) The parameters derived here give the best anion bond-valence sums (RMSD of 0.103 v.u., 5.1% per unit of charge) compared with those obtained from the set of best published parameters (0.120 v.u. and 6.0% per unit of charge) for a set of 122 structures containing 122 unique cations. Furthermore, we conclude that the derivation of bond-valence parameters on the basis of cation coordination polyhedra is sufficient, provided the resultant parameters are verified to work for the anion bond-valence sums *a posteriori*.

## Figures and Tables

**Table 1:** Comparison between the RMSD values (v.u.) of the set of best published bond-valence parameters and the values obtained for bond-valence parameters derived using common methods of derivation for the 90multiple-coordination-number ions

Ion	No. of coordination polyhedra	Best published parameters	Graphical method	Fixing *B* at 0.370	GRG method (this work)
H^+^	224	0.035	0.532	0.040	0.033
Li^+^	690	0.092	0.091	0.115	0.077
Be^2+^	169	0.080	0.092	0.082	0.092
B^3+^	1572	0.069	0.069	0.068	0.069
N^5+^	497	0.162	0.118	0.164	0.118
Na^+^	1683	0.132	0.172	0.157	0.143
Mg^2+^	469	0.120	0.119	0.121	0.110
Al^3+^	856	0.121	0.109	0.115	0.108
Si^4+^	2530	0.126	0.119	0.128	0.119
Cl^3+^	5	0.151	0.374	0.087	0.086
K^+^	1479	0.155	0.212	0.171	0.164
Ca^2+^	1168	0.171	0.174	0.176	0.163
Sc^3+^	88	0.152	0.112	0.140	0.108
Ti^3+^	24	0.183	0.099	0.161	0.094
Ti^4+^	324	0.139	0.155	0.139	0.143
V^3+^	70	0.130	0.113	0.129	0.115
V^4+^	226	0.121	0.109	0.103	0.105
V^5+^	714	0.117	0.105	0.114	0.105
Cr^2+^	17	0.090	0.064	0.060	0.060
Cr^4+^	7	0.242	0.156	0.185	0.156
Mn^2+^	392	0.124	0.124	0.126	0.116
Mn^3+^	94	0.128	0.173	0.130	0.166
Mn^4+^	21	0.122	0.120	0.122	0.120
Fe^2+^	192	0.135	0.115	0.133	0.114
Fe^3+^	466	0.137	0.140	0.138	0.139
Co^2+^	304	0.102	0.099	0.099	0.100
Ni^2+^	255	0.105	0.110	0.107	0.107
Cu^+^	57	0.133	0.081	0.079	0.078
Cu^2+^	716	0.084	0.103	0.085	0.085
Zn^2+^	461	0.085	0.086	0.087	0.085
Ga^3+^	228	0.139	0.139	0.138	0.136
Ge^4+^	350	0.148	0.152	0.148	0.149
As^3+^	28	0.127	0.485	0.086	0.065
As^5+^	526	0.108	0.111	0.109	0.111
Se^4+^	202	0.147	47.223	0.090	0.083
Br^5+^	9	0.147	3.771	0.104	0.064
Rb^+^	464	0.186	0.233	0.171	0.150
Sr^2+^	377	0.222	0.225	0.221	0.189
Y^3+^	178	0.157	0.140	0.157	0.140
Zr^4+^	117	0.135	0.106	0.135	0.106
Nb^5+^	251	0.161	0.162	0.157	0.157
Mo^5+^	76	0.136	0.252	0.116	0.131
Mo^6+^	970	0.147	0.145	0.140	0.143
Ag^+^	200	0.088	0.085	0.080	0.081
Cd^2+^	164	0.122	0.092	0.102	0.088
In^3+^	125	0.200	0.113	0.143	0.111
Sn^2+^	50	0.135	0.125	0.147	0.082
Sn^4+^	38	0.195	0.158	0.196	0.158
Sb^3+^	54	0.085	0.178	0.130	0.084
Te^4+^	212	0.107	4 10^4^	0.108	0.104
I^5+^	134	0.113	108.803	0.130	0.107
I^7+^	36	0.327	0.199	0.212	0.196
Cs^+^	544	0.138	0.176	0.143	0.135
Ba^2+^	857	0.237	0.248	0.231	0.217
La^3+^	182	0.162	0.159	0.153	0.155
Ce^3+^	76	0.162	0.132	0.137	0.131
Ce^4+^	28	0.176	0.124	0.154	0.122
Pr^3+^	99	0.185	0.135	0.146	0.134
Nd^3+^	203	0.160	0.163	0.159	0.159
Sm^3+^	97	0.171	0.149	0.150	0.145
Eu^2+^	3	0.071	0.028	0.047	0.024
Eu^3+^	49	0.196	0.142	0.132	0.134
Gd^3+^	107	0.188	0.141	0.138	0.129
Tb^3+^	48	0.122	0.116	0.117	0.115
Dy^3+^	70	0.174	0.134	0.134	0.129
Ho^3+^	81	0.188	0.129	0.133	0.128
Er^3+^	102	0.141	0.138	0.134	0.133
Tm^3+^	44	0.184	0.146	0.143	0.140
Yb^3+^	82	0.169	0.260	0.172	0.174
Lu^3+^	53	0.175	0.171	0.170	0.162
Hf^4+^	22	0.095	0.087	0.087	0.087
Ta^5+^	162	0.214	0.183	0.185	0.195
W^6+^	436	0.181	0.207	0.182	0.188
Re^7+^	59	0.923	0.192	0.237	0.191
Os^7+^	7		0.230	0.197	0.209
Os^8+^	8	0.608	0.264	0.266	0.233
Ir^4+^	17	0.243	0.136	0.239	0.136
Hg^2+^	52	0.129	0.143	0.120	0.120
Tl^+^	74	0.113	0.101	0.113	0.098
Tl^3+^	9	0.294	0.080	0.145	0.079
Pb^2+^	276	0.125	0.118	0.177	0.111
Pb^4+^	12	0.286	0.184	0.219	0.181
Bi^3+^	231	0.190	0.149	0.152	0.138
Bi^5+^	11	0.316	0.202	0.195	0.203
Th^4+^	27	0.221	0.167	0.182	0.163
U^4+^	18	0.166	0.123	0.116	0.116
U^5+^	4	0.239	0.089	0.214	0.030
U^6+^	585	0.158	0.786	0.226	0.161
Np^5+^	33	0.820	0.126	0.073	0.061
Np^6+^	7	1.209	0.745	0.169	0.083
Weighted mean		0.140	0.161†	0.139	0.128
No. of improvements			59	62	76

† Without the values for Se^4+^, Br^5+^, Te^4+^ and I^5+^ where the method fails.

**Table 2:** RMSD values (v.u.) for a sample of ions for 19 different two-parameter equations fitted with the GRG method

	Equation	Na^+^	Al^3+^	Si^4+^	Ca^2+^	Mn^2+^	Mo^6+^	La^3+^	Pb^2+^	Mean
[1]	*y* = exp[(*a* *x*)/*b*]	0.143	0.108	0.119	0.163	0.116	0.143	0.155	0.111	0.132
[2]	*y* = (*a*+*bx*)^2^	0.127	0.114	0.109	0.164	0.129	0.129	0.180	0.117	0.134
[3]	*y* = [*a*+*b*ln(*x*)]^2^	0.129	0.111	0.113	0.163	0.114	0.131	0.172	0.110	0.130
[4]	*y* = (*a*+*bx* ^0.5^)^2^	0.129	0.112	0.111	0.163	0.119	0.129	0.176	0.113	0.132
[5]	*y* = 1/(*a*+*bx*)	0.136	0.109	0.147	0.174	0.109	0.316	0.158	0.178	0.166
[6]	*y* = 1/(*a*+*bx* ^0.5^)	0.155	0.110	0.151	0.175	0.109	0.339	0.162	0.186	0.173
[7]	*y* = 1/(*a*+*bx* ^1.5^)	0.166	0.109	0.144	0.173	0.109	0.292	0.155	0.170	0.165
[8]	*y* = 1/[*a*+*bx* ^0.5^ln(*x*)]	0.159	0.109	0.148	0.174	0.096	0.322	0.159	0.180	0.168
[9]	*y* = 1/[*a*+*b*exp(*x*)]	0.147	0.112	0.160	0.178	0.110	0.410	0.178	0.219	0.189
[10]	*y* = 1/[*a*+*b*exp(*x*)]	0.136	0.108	0.136	0.168	0.111	0.233	0.147	0.140	0.147
[11]	*y* = exp(*a*+*bx* ^0.5^)	0.133	0.108	0.122	0.163	0.115	0.150	0.153	0.114	0.132
[12]	*y* = exp[*a*+*b*ln(*x*)]	0.135	0.107	0.125	0.164	0.114	0.159	0.151	0.118	0.134
[13]	*y* = exp[*a*+*b*exp(*x*)]	0.139	0.107	0.129	0.168	0.112	0.178	0.147	0.136	0.139
[14]	*y* = [*a*+*b*exp(x)]^2^	0.132	0.109	0.117	0.162	0.115	0.136	0.160	0.110	0.130
[15]†	*y* = *a*+*b*exp(*x*)	0.128	0.117	0.107	0.165	0.118	0.143	0.188	0.126	0.136
[16]†	*y* = *a*+*bx* ^0.5^	0.127	0.123	0.101	0.173	0.123	0.178	0.211	0.168	0.151
[17]†	*y* = *a*+*b*ln(*x*)	0.127	0.120	0.104	0.170	0.121	0.165	0.206	0.157	0.146
[18]‡	*y* = *s*_0_(*a*/*x*)^*b*^	0.135	0.107	0.125	0.164	0.114	0.159	0.151	0.118	0.134
[19]	*y* = *a*/*x* ^2^+*b*/*r* ^3^	0.132	0.109	0.121	0.162	0.114	0.140	0.167	0.109	0.132

† Has an external parameter.

‡ Added manually.

**Table 3:** RMSD values (v.u.) for a sample of ions for seven different three-parameter equations fitted with the GRG method

	Equation	Na^+^	Al^3+^	Si^4+^	Ca^2+^	Mn^2+^	Mo^6+^	La^3+^	Pb^2+^	Mean
[20]	*y* = exp(*a*+*bx*+*cx* ^2^)	0.127	0.105	0.075	0.159	0.114	0.120	0.146	0.107	0.119
[21]	*y* = (*a*++*cx* ^2^)^2^	0.127	0.104	0.075	0.159	0.110	0.125	0.147	0.108	0.119
[22]	*y* = 1/(*a*+*bx*+*cx* ^2^)	0.126	0.108	0.078	0.159	0.110	0.115	0.146	0.107	0.119
[23]	*y* = (*a*+*cx*)/(1+*bx*)	0.127	0.117	0.117	0.165	0.117	0.129	0.152	0.114	0.130
[24]	*y* = (*a*+*cx* ^2^)/(1+*bx* ^2^)	0.127	0.114	0.117	0.162	0.115	0.130	0.150	0.110	0.128
[25]	*y* = (*a*+*cx* ^0.5^)/(1+*bx* ^0.5^)	0.127	0.118	0.117	0.166	0.119	0.129	0.153	0.117	0.131
[26]†	*y* = (*a*+*b*ln(*x*))^2^+*c*	0.127	0.103	0.161	0.159	0.110	0.129	0.147	0.108	0.130

† Added manually

**Table 4:** New bond-valence parameters derived with the GRG method for ions bonded to O^2^

Ion	No. of coordination polyhedra	*R*_o_ ()	*B* ()	RMSD (v.u.)	Method of interpolation†
H^+^	224	0.918	0.427	0.033	
Li^+^	690	1.062	0.642	0.077	
Be^2+^	169	1.429	0.297	0.092	
B^3+^	1572	1.372	0.357	0.069	
C^4+^	433	1.398	0.399	0.086	3
N^5+^	497	1.492	0.482	0.118	
Na^+^	1683	1.695	0.420	0.143	
Mg^2+^	469	1.608	0.443	0.110	
Al^3+^	856	1.634	0.390	0.108	
Si^4+^	2530	1.624	0.389	0.119	
P^3+^	7	1.655	0.399	0.079	3
P^5+^	3691	1.624	0.399	0.099	3
S^4+^	30	1.643	0.399	0.087	3
S^6+^	906	1.634	0.399	0.111	3
Cl^3+^	5	1.722	0.370	0.086	
Cl^5+^	9	1.703	0.428	0.068	2
Cl^7+^	65	1.669	0.428	0.138	2
K^+^	1479	2.047	0.398	0.164	
Ca^2+^	1168	1.907	0.409	0.163	
Sc^3+^	88	1.780	0.452	0.108	
Ti^3+^	24	1.654	0.545	0.094	
Ti^4+^	324	1.819	0.342	0.143	
V^3+^	70	1.718	0.412	0.115	
V^4+^	226	1.776	0.364	0.105	
V^5+^	714	1.799	0.388	0.105	
Cr^2+^	17	1.761	0.350	0.060	
Cr^3+^	104	1.725	0.361	0.114	1
Cr^4+^	7	1.783	0.410	0.156	
Cr^5+^	1	1.777	0.375		2
Cr^6+^	169	1.799	0.375	0.146	2
Mn^2+^	392	1.740	0.417	0.116	
Mn^3+^	94	1.823	0.247	0.166	
Mn^4+^	21	1.750	0.374	0.120	
Mn^5+^	8	1.781	0.375	0.091	2
Mn^6+^	2	1.814	0.375	0.118	2
Mn^7+^	7	1.819	0.375	0.121	2
Fe^2+^	192	1.658	0.447	0.114	
Fe^3+^	466	1.766	0.360	0.139	
Co^2+^	304	1.698	0.376	0.100	
Co^3+^	15	1.655	0.364	0.100	1
Co^4+^	1	1.729	0.358		1
Ni^2+^	255	1.689	0.347	0.107	
Ni^4+^	5	1.734	0.335	0.040	1
Cu^+^	57	1.601	0.335	0.078	
Cu^2+^	716	1.687	0.355	0.085	
Cu^3+^	11	1.737	0.375	0.137	2
Zn^2+^	461	1.684	0.383	0.085	
Ga^3+^	228	1.736	0.345	0.136	
Ge^4+^	350	1.750	0.363	0.149	
As^3+^	28	1.775	0.423	0.065	
As^5+^	526	1.765	0.352	0.111	
Se^4+^	202	1.805	0.401	0.083	
Se^6+^	191	1.797	0.399	0.104	3
Br^5+^	9	1.890	0.571	0.064	
Br^7+^	2	1.850	0.428	0.052	2
Rb^+^	464	1.993	0.478	0.150	
Sr^2+^	377	1.958	0.479	0.189	
Y^3+^	178	1.978	0.407	0.140	
Zr^4+^	117	1.913	0.406	0.106	
Nb^4+^	3	1.853	0.479	0.048	1
Nb^5+^	251	1.909	0.369	0.157	
Mo^3+^	5	1.792	0.436	0.053	1
Mo^4+^	9	1.834	0.404	0.053	1
Mo^5+^	76	1.888	0.314	0.131	
Mo^6+^	970	1.903	0.349	0.143	
Tc^7+^	6	1.915	0.375	0.070	2
Ru^3+^	3	1.745	0.401	0.004	1
Ru^4+^	8	1.833	0.366	0.121	1
Ru^5+^	23	1.894	0.346	0.156	1
Rh^3+^	11	1.769	0.369	0.162	1
Rh^4+^	3	1.836	0.422	0.088	1
Pd^2+^	29	1.749	0.375	0.104	2
Pd^4+^	2	1.856	0.352	0.038	1
Ag^+^	200	1.875	0.359	0.081	
Cd^2+^	164	1.827	0.430	0.088	
In^3+^	125	1.823	0.459	0.111	
Sn^2+^	50	1.910	0.451	0.082	
Sn^4+^	38	1.946	0.274	0.158	
Sb^3+^	54	1.932	0.435	0.084	
Sb^5+^	183	1.892	0.475	0.167	1
Te^4+^	212	1.960	0.389	0.104	
Te^6+^	155	1.922	0.387	0.208	2
I^5+^	134	1.992	0.474	0.107	
I^7+^	36	1.930	0.299	0.196	
Cs^+^	544	2.296	0.411	0.135	
Ba^2+^	857	2.223	0.406	0.217	
La^3+^	182	2.179	0.359	0.155	
Ce^3+^	76	2.114	0.389	0.131	
Ce^4+^	28	2.046	0.416	0.122	
Pr^3+^	99	2.071	0.411	0.134	
Nd^3+^	203	2.103	0.371	0.159	
Sm^3+^	97	2.049	0.404	0.145	
Eu^2+^	3	1.943	0.490	0.024	
Eu^3+^	49	2.068	0.359	0.134	
Gd^3+^	107	1.988	0.433	0.129	
Tb^3+^	48	2.020	0.379	0.115	
Tb^4+^	7	2.018	0.395	0.069	2
Dy^3+^	70	2.002	0.389	0.129	
Ho^3+^	81	1.993	0.387	0.128	
Er^3+^	102	1.991	0.373	0.133	
Tm^3+^	44	1.977	0.381	0.140	
Yb^3+^	82	1.969	0.373	0.174	
Lu^3+^	53	1.939	0.403	0.162	
Hf^4+^	22	1.923	0.375	0.087	
Ta^5+^	162	1.916	0.343	0.195	
W^5+^	4	1.848	0.553	0.128	1
W^6+^	436	1.909	0.339	0.188	
Re^5+^	3	1.834	0.557	0.033	1
Re^7+^	59	1.943	0.406	0.191	
Os^5+^	4	1.870	0.485	0.045	1
Os^6+^	1	1.904	0.375		2
Os^7+^	7	1.937	0.349	0.209	
Os^8+^	8	1.966	0.405	0.233	
Ir^3+^	1	1.755	0.414		1
Ir^4+^	17	1.909	0.258	0.136	
Ir^5+^	6	1.909	0.449	0.138	1
Pt^2+^	3	1.742	0.375	0.040	2
Pt^4+^	33	1.856	0.407	0.136	1
Au^3+^	24	1.890	0.375	0.095	2
Hg^2+^	52	1.947	0.370	0.120	
Tl^+^	74	2.063	0.422	0.098	
Tl^3+^	9	1.874	0.504	0.079	
Pb^2+^	276	2.032	0.442	0.111	
Pb^4+^	12	2.056	0.280	0.181	
Bi^3+^	231	2.068	0.389	0.138	
Bi^5+^	11	2.050	0.318	0.203	
Th^4+^	27	2.117	0.420	0.163	
U^4+^	18	2.100	0.373	0.116	
U^5+^	4	2.009	0.660	0.030	
U^6+^	585	2.046	0.473	0.161	
Np^5+^	33	2.036	0.411	0.061	
Np^6+^	7	2.022	0.523	0.083	
Np^7+^	2	2.076	0.477	0.132	2
Am^3+^	1	2.068	0.392		1
Cm^3+^	1	2.034	0.412		1

† 1: *R*_o_ fixed to predicted value; 2: *B* fixed to family average; 3: *B* fixed to 0.399.

**Table 5:** Overall RMSD (v.u.) for the anion bond-valence sums of four large sets of bond-valence parameters

No. of structures (coordination polyhedra)	Brown Altermatt (1985 ▸)	Brese O’Keeffe (1991 ▸)	Best published parameters	This work
72 (296)	0.130	0.119	0.100	0.100
100 (398)		0.121	0.106	0.099
122 (490)			0.120	0.103
128 (511)				0.104
